# Association of Total Atherosclerosis Burden of Baroreceptor-Resident Arteries with Heart Rate Variability and Its Circadian Rhythm in Patients with Ischemic Stroke

**DOI:** 10.3390/biomedicines14102376

**Published:** 2026-10-18

**Authors:** Yaqin Gu, Luguang Li, Chenxia Zhou, Xiangying Du, Chengbei Hou, Miao Liu, Xin Ma

**Affiliations:** 1 Department of Neurology, Xuanwu Hospital, Capital Medical University, Beijing 100053, China; 2 National Clinical Research Center for Geriatric Diseases, Beijing 100053, China; 3 Department of Radiology, Xuanwu Hospital, Capital Medical University, Beijing 100053, China; 4 Center for Evidence-Based Medicine, Xuanwu Hospital, Capital Medical University, Beijing 100053, China; 5 Department of Cardiology, Xuanwu Hospital, Capital Medical University, Beijing 100053, China

**Keywords:** ischemic stroke, atherosclerosis, baroreceptor-resident arteries, heart rate variability, circadian rhythm, cardiac autonomic function

## Abstract

**Background/Objectives**: Post-stroke cardiac autonomic dysfunction is associated with poor neurological recovery and cardiovascular events. However, its association with vascular disease remains incompletely understood. We evaluated associations of atherosclerotic burden in baroreceptor-resident arteries (BRAs), including the carotid sinus (CS) and aortic arch (AA), with heart rate variability (HRV) and its circadian rhythm after ischemic stroke. **Methods:** This cross-sectional study consecutively enrolled 253 patients with first-ever ischemic stroke within 30 days of onset. Total atherosclerosis burden of baroreceptor-resident arteries (TAB-BRA) was assessed using computed tomography angiography (CTA), and patients were categorized based on TAB-BRA tertiles. The 24 h HRV indices and serial measures across 48 consecutive 30 min segments were derived from Holter recordings, with the standard deviation of all normal-to-normal intervals (SDNN) as the primary outcome. **Results:** After adjusting for age, sex, hypertension, stroke severity, time from stroke onset to Holter monitoring, and other prespecified covariates, each 1-point increase in TAB-BRA was associated with a 1.67 ms decrease in 24 h SDNN (95% confidence interval [CI], −2.43 to −0.90; *p* < 0.001), suggesting impaired cardiac autonomic regulation. Restricted cubic spline models revealed a nonlinear association (*p* for nonlinearity = 0.014). Adding AA burden to the model containing CS burden improved model fit for 24 h SDNN (adjusted R2 difference = 0.033; *p* = 0.001). Compared with Tertile 1, circadian SDNN amplitude was lower by 3.61 ms in Tertile 2 (95% CI, −6.33 to −0.81; *p* = 0.006) and 3.71 ms in Tertile 3 (95% CI, −6.59 to −0.77; *p* = 0.012). **Conclusions:** Higher TAB-BRA was nonlinearly associated with lower 24 h SDNN, while the intermediate- and high-burden groups had lower circadian SDNN amplitudes. CTA-based TAB-BRA assessment may provide complementary information for evaluating post-stroke cardiac autonomic dysfunction.

## 1. Introduction

Cardiac autonomic dysfunction is common after ischemic stroke and is associated with poor long-term neurological recovery and an increased risk of cardiovascular events [1,2]. Beyond ischemic injury to the central autonomic network [3], vascular factors also play a critical role in cardiac autonomic dysfunction, yet this relationship remains incompletely understood [4]. Heart rate variability (HRV) is a well-established, non-invasive measure derived from fluctuations in beat-to-beat sinus intervals across different timescales, including long-term 24 h and short-term 5 min recordings [5]. It provides a standardized method to evaluate cardiac autonomic modulation and to investigate its relationship with vascular disease after stroke.

The arterial baroreflex represents a crucial feedback loop through which vascular properties influence cardiac autonomic regulation [6]. Arterial baroreceptors are located primarily in the carotid sinus (CS) and aortic arch (AA). Atherosclerotic plaques in these baroreceptor-resident arteries (BRAs) may reduce local arterial compliance and distensibility, impairing baroreceptor afferent signaling and cardiac autonomic regulation [7]. Theoretically, BRA atherosclerosis may be differentially associated with HRV indices that reflect distinct aspects of autonomic regulation, although the specific patterns of these associations remain unclear. Previous studies have focused on ultrasonographic assessment of atherosclerotic burden in the carotid territory alone [8]. By contrast, the deep anatomical location of the aortic arch limits direct and precise quantification of its atherosclerotic burden using conventional ultrasonography, leaving this territory relatively understudied in relation to cardiac autonomic regulation. To address this limitation, our team previously established a total atherosclerosis burden of baroreceptor-resident arteries (TAB-BRA) score based on combined head and neck computed tomography angiography (CTA), integrating plaque involvement across both regions [9,10]. The previous study provided preliminary evidence suggesting correlations between BRA atherosclerosis and certain HRV indices [9]. However, that study relied primarily on correlation analyses, which could not quantify changes in HRV parameters associated with TAB-BRA, and did not systematically characterize the detailed pattern of association between the atherosclerotic burden of BRAs and HRV, including its dose–response relationship and potential effect modification by clinical characteristics. Moreover, to date, direct evidence comparing the associations of atherosclerotic burden in these two vascular beds with cardiac autonomic function remains lacking, and whether aortic arch atherosclerotic burden provides incremental explanatory value beyond carotid sinus atherosclerotic burden has not been established.

Beyond the overall level of cardiac autonomic modulation assessed by 24 h HRV indices, serial short-term measurements can capture its circadian rhythm, which typically exhibits lower daytime levels and higher nocturnal values, reflecting physiological day–night variations in autonomic tone [11,12]. Previous studies have reported an attenuated circadian rhythm of HRV after stroke [13] and associations between abnormal HRV rhythmic fluctuations and recurrent ischemic events [14]. Cosinor models provide a framework for characterizing these 24 h trajectories using the midline estimating statistic of rhythm (MESOR), amplitude, and acrophase (peak time), which represent the rhythm-adjusted mean, magnitude of rhythmic variation, and timing of the fitted peak, respectively [15]. Compared with conventional day/night segmentation, which reduces continuous 24 h variation to two predefined time windows, mixed-effects cosinor modeling preserves the temporal structure of repeated HRV measurements and enables quantitative characterization of circadian rhythmicity. However, whether the atherosclerotic burden of the BRAs is associated with further disruption of HRV rhythmicity after stroke has not been systematically investigated.

This study therefore aimed to characterize the pattern of association between the atherosclerotic burden of the BRAs and 24 h HRV indices in patients with ischemic stroke, while determining the incremental explanatory value of the aortic arch beyond carotid sinus assessment. We further examined the associations between TAB-BRA and circadian rhythm features of HRV. These analyses were intended to provide further insight into vascular factors and potential mechanisms underlying post-stroke cardiac autonomic dysfunction. We hypothesized that a higher TAB-BRA score would be associated with reduced overall HRV and blunted circadian amplitude, and that aortic arch burden would provide incremental explanatory value over carotid sinus burden alone.

## 2. Materials and Methods

### 2.1. Study Design and Participants

This was a cross-sectional study based on a single-center registry cohort. We consecutively screened patients with ischemic stroke who were admitted to the stroke unit of the Department of Neurology, Xuanwu Hospital, Capital Medical University, between April 2022 and November 2025. The study was conducted in accordance with the Declaration of Helsinki and was approved by the Ethics Committee of Xuanwu Hospital, Capital Medical University (approval No. [2022]008). All enrolled participants provided written informed consent.

The inclusion criteria were as follows: (1) age 18–85 years; (2) first-ever ischemic stroke confirmed by clinical assessment and cranial magnetic resonance imaging (MRI); (3) enrollment within 30 days of stroke onset; (4) completion during hospitalization of CTA covering the carotid sinus and aortic arch; (5) completion of 24 h Holter monitoring; (6) complete clinical data; and (7) provision of written informed consent.

The exclusion criteria were as follows: (1) a history of ischemic stroke, hemorrhagic stroke, or subarachnoid hemorrhage; (2) conditions that could affect autonomic function, such as thyroid dysfunction, Parkinson disease, multiple system atrophy, or pheochromocytoma; (3) use of medications that could affect autonomic function, such as β-blockers, antiarrhythmic agents, tricyclic antidepressants, selective serotonin reuptake inhibitors, or anticholinergics; (4) severe infection, active malignancy, or severe electrolyte disturbance; (5) worsening neurologic deficits within the 3 days before Holter monitoring; (6) a contraindication to or inability to tolerate CTA; (7) previous surgery involving the carotid sinus or aortic arch; (8) carotid or aortic dissection; (9) fewer than 20 h of valid Holter recording; (10) atrial fibrillation or atrial flutter during monitoring; and (11) more than 5% of beats being unanalyzable because of frequent premature atrial or ventricular contractions, high-grade atrioventricular block, artifacts, or other causes.

### 2.2. Assessment of TAB-BRA

All participants underwent single-session CTA of the head, neck, and aortic arch using a third-generation dual-source computed tomography (CT) scanner (Somatom Force; Siemens Healthcare, Munich, Germany), with scan coverage extending from the base of the aortic arch to the cranial vertex. Images were reconstructed and analyzed using Syngo.Via software (Version VB50A; Siemens Healthcare, Erlangen, Germany).

Atherosclerotic burden in the BRAs was assessed semiquantitatively using the TAB-BRA score, as described previously [9,10]. The score encompasses 10 vascular segments across the carotid sinus and aortic arch. The CS region comprises the bilateral distal common carotid arteries, bilateral carotid bifurcations, and bilateral proximal internal carotid arteries (6 segments). The AA region comprises the aortic arch—from the opening of the brachiocephalic trunk to the opening of the left subclavian artery—and the origins of the brachiocephalic trunk, left common carotid artery, and left subclavian artery (4 segments). For each segment, the proportion of the vessel circumference affected by plaque was assessed on an orthogonal cross-sectional image perpendicular to the vessel’s long axis and scored as follows: 0, no visible lesion; 1, <25% of the circumference; 2, 25–49%; 3, 50–74%; and 4, ≥75%. Segment scores were summed to yield the total TAB-BRA score (range, 0–40), with higher scores indicating a greater atherosclerotic burden in the BRAs. The atherosclerotic burden of the aortic arch (AB-AA; range, 0–16) and the atherosclerotic burden of the carotid sinus (AB-CS; range, 0–24) were also calculated separately.

CTA images were independently evaluated and TAB-BRA scores assigned by 2 trained neurologists who were blinded to the clinical data. Discrepancies were resolved by consensus between the two readers, with adjudication by a neuroradiology expert when necessary. Inter-rater reliability of the pre-consensus total TAB-BRA scores was assessed using a two-way mixed-effects model with absolute agreement and single-measure intraclass correlation coefficient (ICC) [16]. Based on tertiles of the total TAB-BRA score, participants were classified into a low-burden group (Tertile 1; TAB-BRA, 0–1), an intermediate-burden group (Tertile 2; TAB-BRA, 2–5), and a high-burden group (Tertile 3; TAB-BRA, 6–40).

### 2.3. Holter Monitoring for 24 H and HRV Assessment

After the neurologic condition had remained stable for 3 days, participants underwent continuous 24 h 12-lead Holter monitoring using a DMS 300-4AL ambulatory electrocardiographic recorder (DM Systems [Beijing] Co., Ltd., Beijing, China) at a sampling rate of 1024 Hz. After monitoring, the recordings were imported into the accompanying CardioScan Holter analysis software (Version 12, DM Systems [Beijing] Co., Ltd., Beijing, China) and manually reviewed by a physician with expertise in electrocardiography.

HRV processing and calculation followed the expert consensus issued by the Task Force of the European Society of Cardiology and the North American Society of Pacing and Electrophysiology [5], together with a prespecified quality control protocol. The raw R–R interval series was first screened to exclude physiologically implausible intervals (<400 ms or >2000 ms). Artifacts, ectopic beats, and non-sinus beats were identified and removed, leaving only normal-to-normal (NN) intervals between sinus beats. Isolated abnormal intervals that could be corrected reliably were reconstructed by cubic spline interpolation to obtain an evenly sampled time series. Power spectral density was then calculated using the fast Fourier transform to derive frequency-domain indices. A recording was considered invalid and excluded if the valid recording time was <20 h or if >5% of beats were unanalyzable.

Using the NN intervals retained after quality control, HRV indices were calculated for the whole 24 h recording, daytime (06:00–22:00), and nighttime (22:00–06:00 the following day).

For circadian rhythm analyses, as Holter monitoring was routinely initiated at approximately 09:00 at our center, recordings were aligned to the actual clock time of 09:00, and each 24 h analysis cycle was defined from 09:00 to 09:00 the following day. Each Holter recording was first divided into 288 consecutive 5 min periods, and HRV parameters were calculated for each period. Parameters from every six adjacent 5 min periods were averaged into 30 min intervals, yielding 48 consecutive HRV measures per participant for circadian rhythm analysis. It should be noted that HRV parameters derived from recordings of different durations are not directly comparable.

HRV measures included time- and frequency-domain indices. Time-domain indices comprised the standard deviation of all normal-to-normal intervals (SDNN) and the root mean square of successive differences between adjacent NN intervals (rMSSD). Frequency-domain indices comprised low-frequency power (LF; 0.04–0.15 Hz) and high-frequency power (HF; 0.15–0.40 Hz). LF and HF were natural log-transformed before analysis and are reported as log LF and log HF, respectively.

SDNN was prespecified as the primary outcome and was used to represent overall HRV. The rMSSD, log LF, and log HF were prespecified as secondary outcomes.

### 2.4. Clinical Data Collection

Baseline data included demographic characteristics (age, sex, and body mass index [BMI]), vascular risk factors (hypertension, diabetes mellitus, and smoking status), medical history, and medication use. Neurologic deficit severity on admission was assessed using the National Institutes of Health Stroke Scale (NIHSS), and the interval from stroke onset to the start of Holter monitoring was recorded. Infarct location was determined using cranial MRI, with insular and brainstem involvement recorded separately.

### 2.5. Statistical Analysis

All statistical analyses were performed using R version 4.4.1 (R Foundation for Statistical Computing, Vienna, Austria), with packages including cosinoRmixedeffects (Version 0.1.0) [17]. All tests were two-sided, and *p* < 0.05 was considered statistically significant. Normally distributed continuous variables are presented as mean ± standard deviation and were compared across groups using one-way analysis of variance. Non-normally distributed continuous variables are presented as median (interquartile range [IQR]) and were compared using the Kruskal–Wallis test. Categorical variables are presented as number (percentage) (*n* [%]) and were compared using the Pearson χ^2^ test or Fisher exact test, as appropriate.

Multivariable linear regression models were used to evaluate the associations of TAB-BRA with HRV indices over 24 h, during daytime, and during nighttime. Covariates were prespecified on the basis of prior evidence and clinical relevance and included age, sex, hypertension, diabetes mellitus, BMI, history of coronary artery disease, smoking status, time from stroke onset to Holter monitoring, NIHSS score, insular involvement, and brainstem involvement. All patients included in the final analyses had complete data for all prespecified covariates, and no missing data imputation was performed. Regression coefficients (β) and 95% confidence intervals (CIs) provided summary linear estimates of the change in an HRV index per 1-point increase in atherosclerotic burden across the observed score range. SDNN was prespecified as the primary outcome, whereas the other HRV indices were prespecified as secondary outcomes. Analyses of secondary outcomes were not adjusted for multiple comparisons.

To assess the incremental value of AB-AA beyond AB-CS in improving in-sample model fit, nested multivariable linear regression models were constructed. The base model included AB-CS and the prespecified covariates, whereas the full model additionally included AB-AA. Changes in model fit were summarized using adjusted R^2^, and the nested models were compared using a partial F test. Akaike Information Criterion (AIC) was additionally calculated as a complementary measure of model fit, with lower values indicating better fit. To compare the associations of AB-AA and AB-CS with HRV, both scores were standardized to Z scores and entered simultaneously into the multivariable linear regression model. Regression coefficients and 95% CIs were estimated for each HRV outcome per 1 standard deviation (SD) increase in each score. The between-coefficient difference and its 95% CI were also estimated.

Restricted cubic spline (RCS) models were further used to evaluate nonlinear associations between TAB-BRA and the HRV indices, with 4 knots placed at the 5th, 35th, 65th, and 95th percentiles of the TAB-BRA distribution, following the knot placement strategy recommended by Harrell [18]. Models were adjusted for all prespecified covariates, and Wald tests were used to assess the overall association and nonlinearity. Prespecified subgroup analyses were performed for 24 h SDNN, with interaction terms used to evaluate potential effect modification. The age cutoff of 60 years was prespecified based on previous evidence of age-related changes in cardiac autonomic regulation and its use in HRV studies of patients with ischemic stroke [19,20].

Mixed-effects cosinor models were fitted to serial 30 min HRV measurements to account for repeated measurements within participants and between-participant variation in rhythmic patterns. A fixed 24 h period was assumed. Rhythm parameters included the MESOR, amplitude, and peak time, representing the rhythm-adjusted mean, half the peak-to-trough difference in the fitted curve, and the clock time of the fitted peak, respectively.

For participant *i*, the model was expressed as

$$Y_{i}\left(t\right)=M_{i}+A_{i}\cdot \cos\left[\frac{2\pi }{24}\cdot \left(t-\varphi_{i}\right)\right]+\varepsilon_{\mathrm{it}\;}\;$$

For estimation, this equation was reparameterized as

$$Y_{i}\left(t\right)=M_{i}+\beta_{\mathrm{ci}}\cdot \cos\left(\frac{2\pi }{24}t\right)+\beta_{\mathrm{si}}\cdot \sin\left(\frac{2\pi }{24}t\right)+\varepsilon_{\mathrm{it}}$$

where *t* denotes clock time in hours, *Y_i_(t)* is the HRV outcome on the analysis scale, *M_i_* is the participant-specific MESOR, *β_ci_* and *β_si_* are the participant-specific cosine and sine coefficients, and *ε_it_* is the residual error. Amplitude and peak time were derived as

$$A_{i}=\sqrt{\beta_{\mathrm{ci}}^{2}+\beta_{\mathrm{si}}^{2}}$$

Peak time *φ_i_* was treated on a circular 24 h scale to avoid misinterpretation when a confidence interval crossed midnight and was calculated as follows:

$$\varphi_{i}=\left[\frac{24}{2\pi }\cdot \mathrm{atan}2\left(\beta_{\mathrm{si}},\beta_{\mathrm{ci}}\right)\right]\;\mathrm{mod}\;24$$

TAB-BRA was modeled separately as a continuous variable and as a tertile-based categorical variable. Fixed effects included TAB-BRA, the sine and cosine terms, the prespecified covariates, and the corresponding interaction terms. At the participant level, the models included a random intercept and random slopes for the sine and cosine terms to capture between-participant variation in rhythmic patterns. Because amplitude and peak time are nonlinear combinations of fixed-effect coefficients, a parametric bootstrap with 2000 simulations (random seed, 20260702) was used to derive marginal estimates based on the fixed effects, along with the corresponding standard errors and 95% CIs.

## 3. Results

### 3.1. Baseline Characteristics

**Figure 1:**
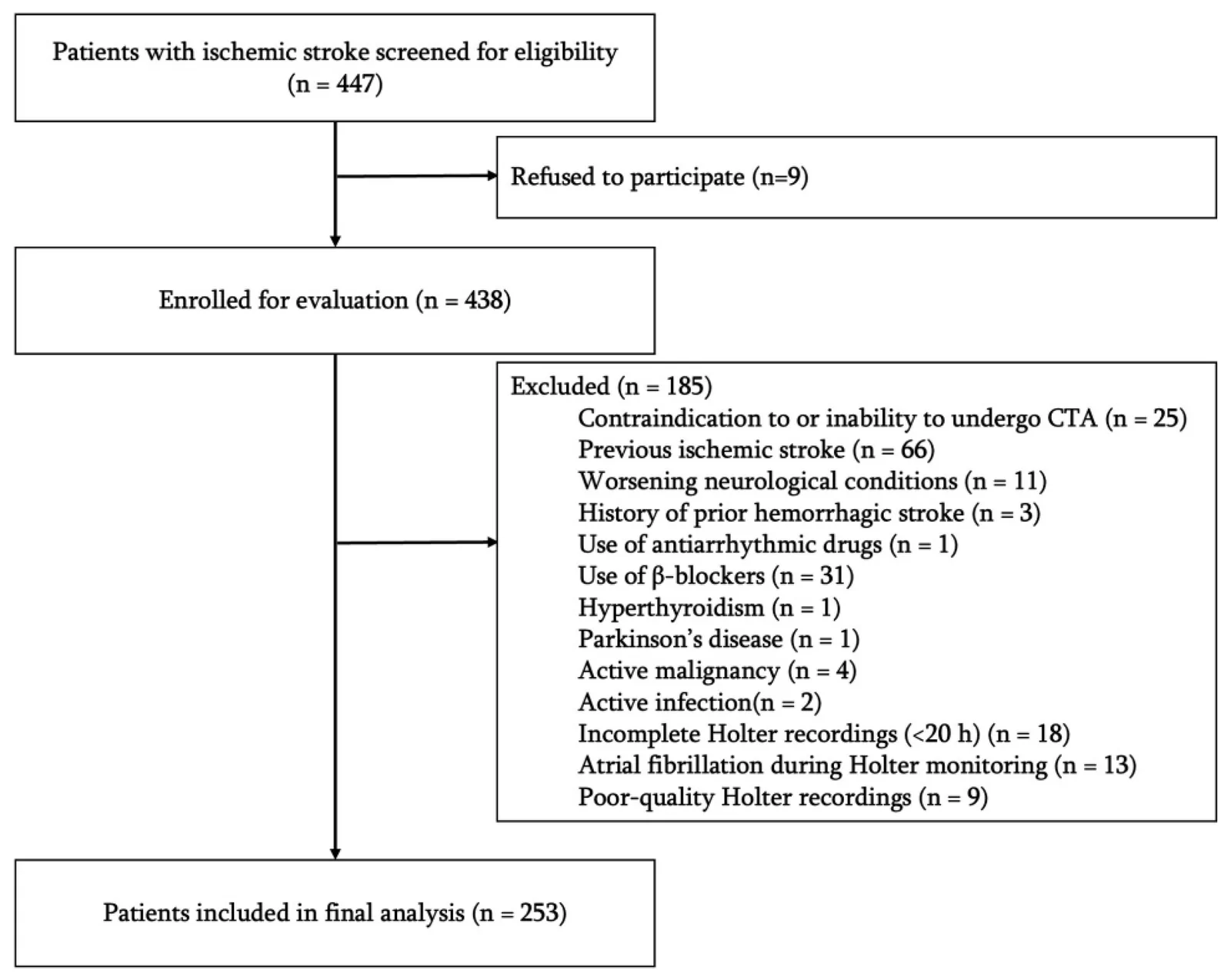
Flowchart of patient screening. Abbreviation: CTA, computed tomography angiography.

A total of 447 patients with ischemic stroke were initially screened. Of these, nine declined to provide written informed consent, leaving 438 for further assessment. After an additional 185 patients were excluded according to the prespecified exclusion criteria, 253 patients were included in the final analysis (Figure 1).

The median age of the overall cohort was 60 years (IQR, 52–67 years), and 187 patients (73.9%) were men. The median NIHSS score at admission was 2 (IQR, 1–4), and the median interval from stroke onset to Holter monitoring was 7 days (IQR, 5–11). For the inter-rater reliability of TAB-BRA assessment, the ICC for the pre-consensus total TAB-BRA score was 0.875 (95% CI, 0.798–0.924) in the enrolled patients. When patients were grouped by TAB-BRA tertile, 75, 85, and 93 patients were assigned to Tertile 1, Tertile 2, and Tertile 3, respectively. Patients with a higher TAB-BRA burden were older and more likely to have hypertension and a history of smoking; systolic blood pressure also differed across the groups. No statistically significant between-group differences were observed in the remaining demographic or clinical baseline characteristics (Table 1).

In unadjusted between-group comparisons (Table 1 and Supplementary Table S1), SDNN, rMSSD, LF, and HF were each progressively lower across increasing tertiles, with overall and trend *p* values < 0.05 for all four indices. Similar patterns were observed for daytime and nighttime HRV parameters.

### 3.2. Association Between TAB-BRA and 24 H HRV Parameters

After adjustment for the prespecified covariates, multivariable linear regression showed that higher TAB-BRA was associated with lower 24 h SDNN and log LF (Table 2). Across the observed TAB-BRA range, the linear models yielded summary estimates of a 1.67 ms decrease in 24 h SDNN (95% CI, −2.43 to −0.90; *p* < 0.001) and a 0.05-unit decrease in 24 h log LF (95% CI, −0.07 to −0.03; *p* < 0.001) per 1-point increase in TAB-BRA. No statistically significant associations were observed for rMSSD or log HF. Daytime and nighttime analyses yielded patterns consistent with those for the 24 h indices (Supplementary Table S2). Given the significant differences in systolic blood pressure across TAB-BRA tertiles, sensitivity analyses were performed by replacing hypertension status with systolic blood pressure as a continuous covariate. The associations between TAB-BRA and 24 h HRV indices remained materially unchanged (Supplementary Table S3).

**Figure 2:**
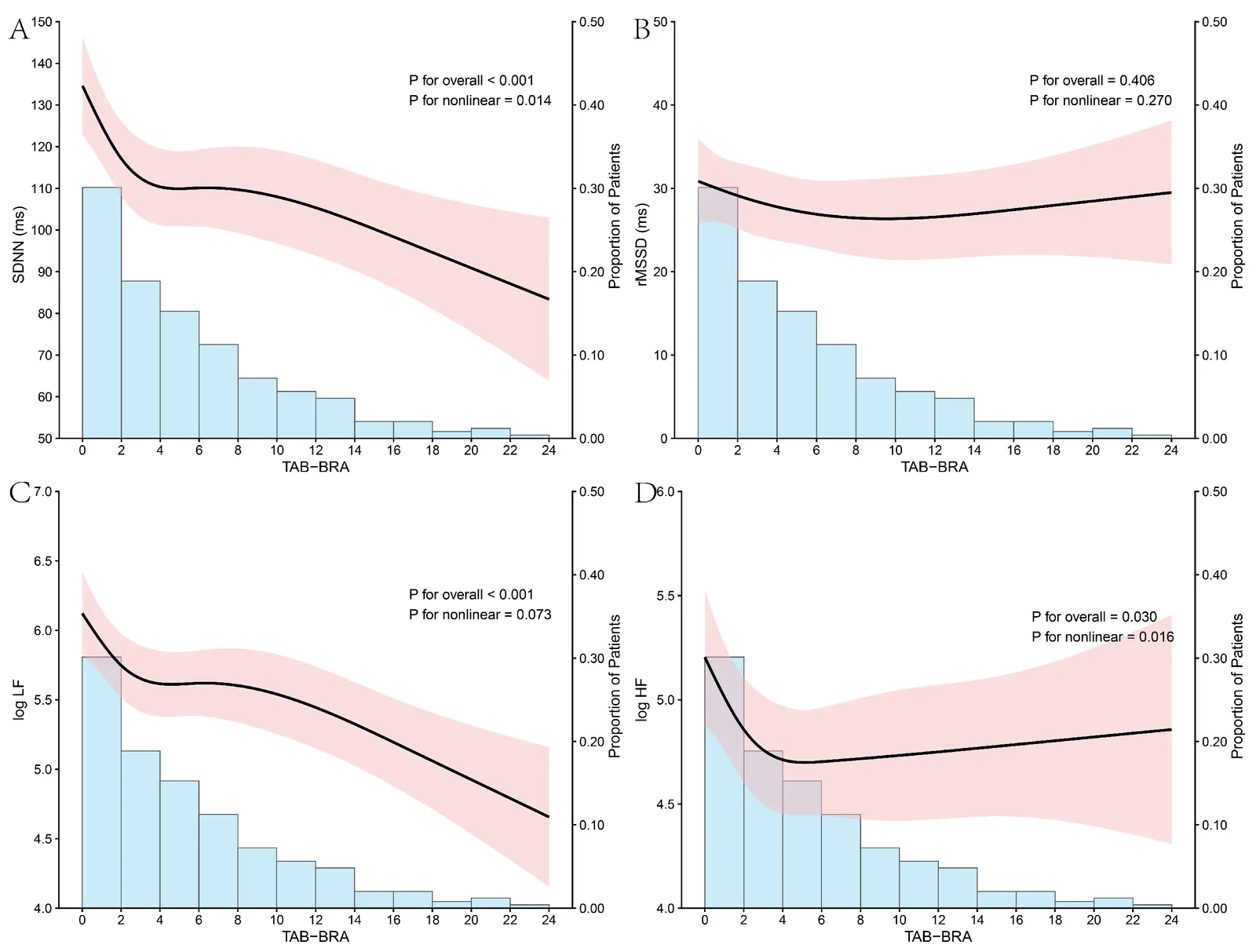
Restricted cubic spline analyses of the associations between TAB-BRA and 24 h HRV parameters. Panels (**A**–**D**) show 24 h SDNN, rMSSD, log LF, and log HF, respectively. Solid black lines represent the fitted restricted cubic spline functions, pink shaded areas represent 95% confidence intervals, and light-blue histograms show the distribution of TAB-BRA scores using the right-hand proportion axis. Four knots were placed at the 5th, 35th, 65th, and 95th percentiles of the TAB-BRA distribution, corresponding to scores of 0, 2, 6, and 16.4, respectively. Models were adjusted for age, sex, hypertension, diabetes mellitus, body mass index, history of coronary artery disease, smoking, time from stroke onset to Holter monitoring, National Institutes of Health Stroke Scale, insular involvement, and brainstem involvement. Abbreviations: TAB-BRA, total atherosclerosis burden of baroreceptor-resident arteries; HRV, heart rate variability; SDNN, standard deviation of all normal-to-normal intervals; rMSSD, root mean square of successive differences between adjacent normal-to-normal intervals; LF, low-frequency power; HF, high-frequency power.

RCS analysis showed a significant overall association between TAB-BRA and 24 h SDNN (*p* for overall < 0.001), with evidence that the association was nonlinear (*p* for nonlinearity = 0.014; Figure 2). The fitted curve showed a steeper decline at lower TAB-BRA scores, followed by a more gradual decline at moderate-to-high scores. TAB-BRA was also associated with lower 24 h log LF (*p* for overall < 0.001), although the test for nonlinearity was not statistically significant (*p* for nonlinearity = 0.073). A nonlinear association between TAB-BRA and 24 h log HF was observed (*p* for overall = 0.030; *p* for nonlinearity = 0.016). No significant overall association was observed for rMSSD (*p* for overall = 0.406).

**Figure 3:**
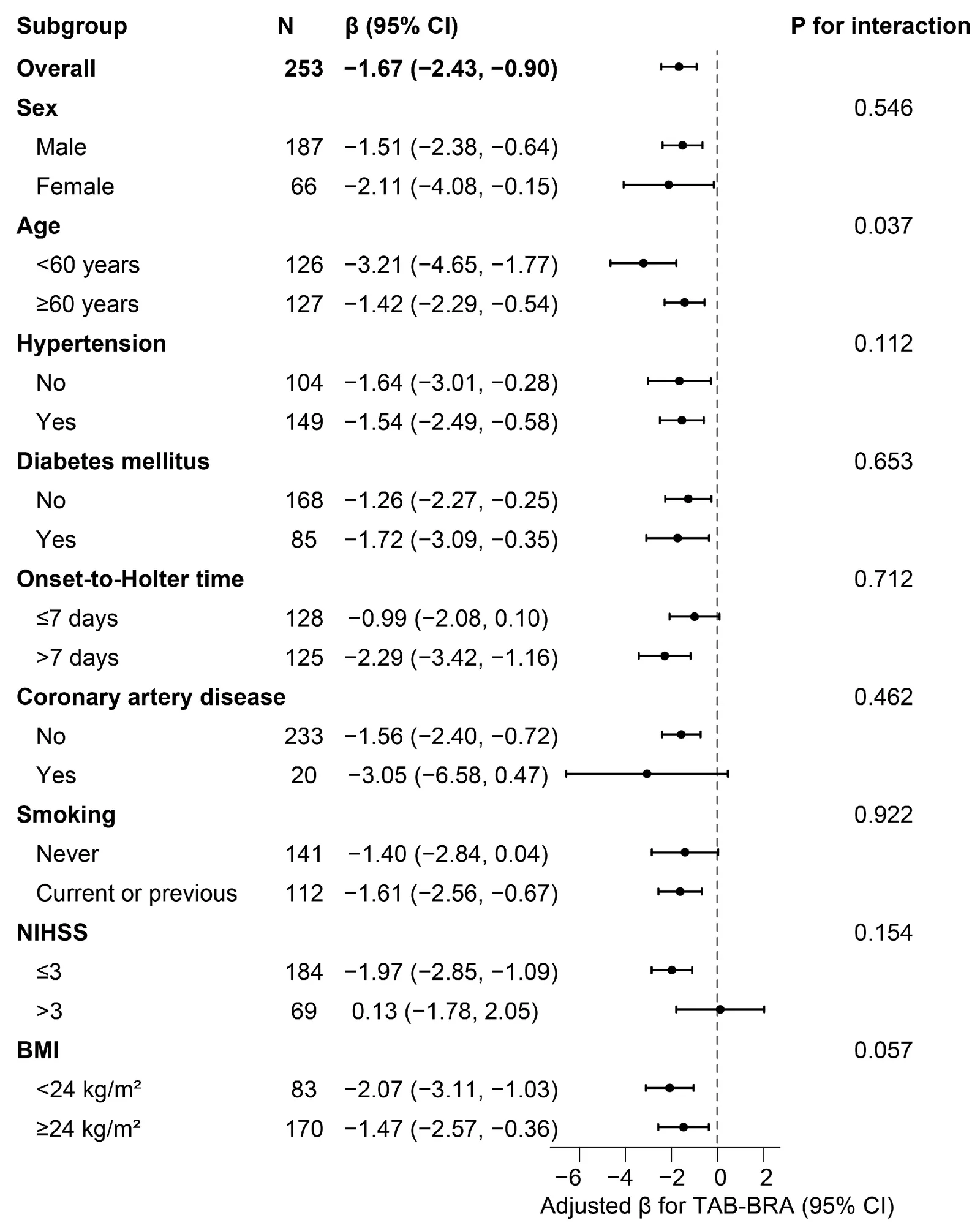
Prespecified subgroup analyses of the association between SDNN and TAB-BRA. Solid circles represent adjusted coefficients for the change in SDNN per 1-point increase in TAB-BRA, and horizontal lines represent 95% confidence intervals. The vertical dashed line denotes the null value (β = 0). *p* for interaction tests whether the association between TAB-BRA and SDNN differs across subgroup levels. N represents number of patients. Models were adjusted for age, sex, hypertension, diabetes mellitus, body mass index, history of coronary artery disease, smoking, time from stroke onset to Holter monitoring, National Institutes of Health Stroke Scale, insular involvement, and brainstem involvement. Abbreviations: BMI, body mass index; NIHSS, National Institutes of Health Stroke Scale; TAB-BRA, total atherosclerosis burden of baroreceptor-resident arteries; SDNN, standard deviation of all normal-to-normal intervals.

Subgroup analyses showed that the inverse association between TAB-BRA and 24 h SDNN was directionally consistent across most clinical subgroups (Figure 3). The *p* for interaction by age was 0.037, with a larger absolute effect estimate among patients aged <60 years. No statistically significant interactions were observed in the other subgroups.

### 3.3. Incremental Value and Site-Specific Associations of Aortic Arch and Carotid Sinus Atherosclerotic Burden

In separate models relating AB-AA and AB-CS to 24 h HRV parameters, higher AB-AA and AB-CS burdens were each associated with lower SDNN and log LF after adjustment for the prespecified covariates (Supplementary Table S4). Adding AB-AA to the base model containing AB-CS and the prespecified covariates significantly improved model fit for 24 h SDNN (adjusted R^2^ difference = 0.033; *p* = 0.001), rMSSD (adjusted R^2^ difference = 0.015; *p* = 0.032), and log HF (adjusted R^2^ difference = 0.012; *p* = 0.040) (Table 3). Consistently, AIC values were lower after inclusion of AB-AA for all four HRV indices (Supplementary Table S5).

When Z-standardized AB-AA and AB-CS were entered simultaneously into the model, the difference between the two site-specific effects on rMSSD was statistically significant (β difference, −3.80; 95% CI, −7.46 to −0.14; *p* = 0.042). For SDNN and log HF, the absolute estimated HRV-lowering effects were generally greater for standardized AB-AA than for standardized AB-CS, but none of these differences reached statistical significance (Table 4).

### 3.4. Association Between TAB-BRA and Circadian Rhythm Parameters of HRV

Mixed-effects cosinor models were used to evaluate associations between TAB-BRA and HRV rhythm parameters. When TAB-BRA was modeled as a continuous variable, each 1-point higher TAB-BRA score was associated with a 0.70 ms lower SDNN MESOR (95% CI, −1.04 to −0.34; *p* < 0.001) after adjustment for the prespecified covariates. The estimated change in SDNN amplitude was −0.18 ms (95% CI, −0.38 to 0.03; *p* = 0.096), which was not statistically significant (Table 5). A higher TAB-BRA score was also associated with a lower log LF MESOR (*p* < 0.001), but not with log LF amplitude (Supplementary Table S7). No statistically significant associations were observed with the MESOR or amplitude of rMSSD or log HF when TAB-BRA was modeled continuously (Supplementary Tables S8 and S9).

**Figure 4:**
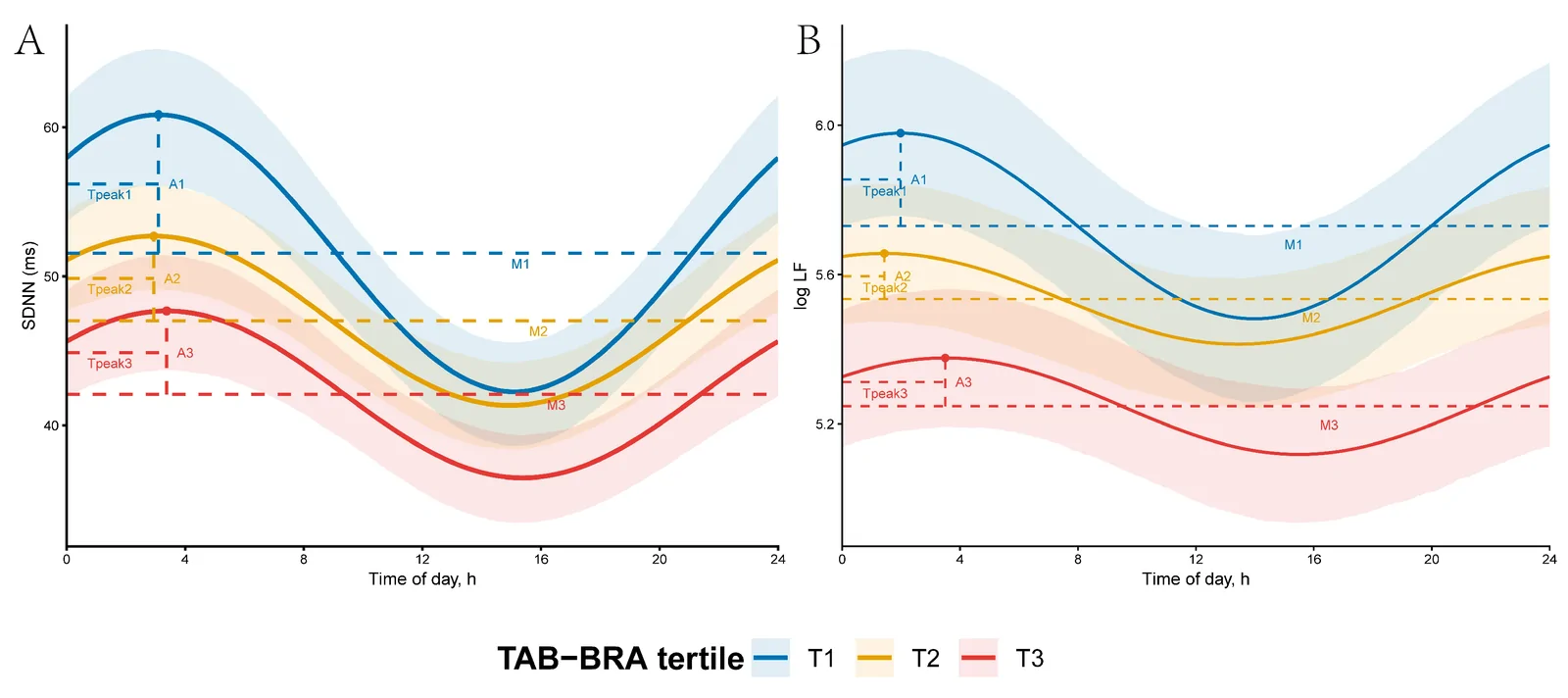
Multivariable-adjusted fitted rhythm curves of SDNN and log LF by TAB-BRA tertiles. Panels (**A**,**B**) show adjusted 24 h rhythms of SDNN and log LF across TAB-BRA Tertiles 1–3, with blue, orange, and red lines representing fitted cosinor curves for Tertiles 1, 2, and 3, respectively, and shaded areas denoting 95% confidence intervals. Long horizontal dashed lines (labeled M1–M3) indicate the MESOR; vertical dashed segments (labeled A1–A3) from the MESOR to the peak indicate amplitude; and the x-coordinates of the peak points (labeled Tpeak1–Tpeak3) indicate peak times for Tertiles 1–3, respectively. Models were adjusted for age, sex, hypertension, diabetes mellitus, body mass index, history of coronary artery disease, smoking, time from stroke onset to Holter monitoring, National Institutes of Health Stroke Scale, insular involvement, and brainstem involvement. Abbreviations: TAB-BRA, total atherosclerosis burden of baroreceptor-resident arteries; SDNN, standard deviation of all normal-to-normal intervals; LF, low-frequency power; MESOR, midline estimating statistic of rhythm.

In analyses by TAB-BRA tertiles, the adjusted SDNN MESOR was progressively lower across increasing tertiles. Compared with Tertile 1, the rhythmic amplitude of SDNN was lower by 3.61 ms in Tertile 2 (95% CI, −6.33 to −0.81; *p* = 0.006) and by 3.71 ms in Tertile 3 (95% CI, −6.59 to −0.77; *p* = 0.012) (Table 5, Figure 4). For log LF, MESOR was significantly lower in Tertile 3, whereas amplitude was lower in both Tertiles 2 and 3 relative to Tertile 1, reaching statistical significance in Tertile 2 (*p* = 0.037) but not in Tertile 3 (*p* = 0.073) (Figure 4, Supplementary Table S7). Both rMSSD and log HF amplitudes were lower in Tertiles 2 and 3 than in Tertile 1 (Supplementary Tables S8 and S9; Figure S1). TAB-BRA was not significantly associated with peak time for any HRV parameter.

## 4. Discussion

The main findings of this study were as follows. First, after adjustment for prespecified covariates, higher TAB-BRA was independently associated with lower 24 h SDNN and log LF, but no significant associations were observed with rMSSD or log HF. Second, model comparisons showed that adding AB-AA to AB-CS significantly improved the explanatory power for 24 h SDNN. Third, RCS analysis revealed a nonlinear inverse dose–response relationship between TAB-BRA and 24 h SDNN, characterized by a steep decline in the low-burden range and relative flattening in the moderate-to-high-burden range. Notably, circadian analysis revealed that the amplitude of SDNN was significantly attenuated in the intermediate and high TAB-BRA tertiles, whereas peak time did not shift significantly. These results suggest that a higher atherosclerotic burden in the BRAs is associated with reduced overall HRV and blunted circadian variation after ischemic stroke.

HRV serves as an important measure of cardiac autonomic regulation that correlates with post-stroke functional recovery and cardiovascular outcomes [2]. In this study, after fully adjusting for covariates, elevated TAB-BRA was independently associated with lower 24 h SDNN, supporting an inverse relationship between the atherosclerotic burden of the BRAs and overall cardiac autonomic modulation and corroborating preliminary findings by Pereira et al. [7] and Fu et al. [9]. Notably, TAB-BRA was significantly associated with log LF but not with rMSSD or log HF. While SDNN represents overall HRV, LF, HF, and rMSSD arise from distinct physiological mechanisms [21]. LF power is modulated by both sympathetic and parasympathetic activity and is closely related to baroreflex-mediated blood pressure–heart rate coupling. Under physiological conditions, arterial blood pressure oscillates periodically at approximately 0.1 Hz, generating heart rate fluctuations within the LF band via baroreceptor feedback [22]. Conversely, rMSSD and HF predominantly reflect short-term vagal modulation driven by respiration. As suggested by previous studies in which baroreflex sensitivity (BRS) was used to directly assess baroreflex function, atherosclerotic lesions in the CS and AA may reduce arterial compliance and distensibility, attenuating the transmission of pulsatile pressure into vessel wall stretch and thereby impairing baroreceptor afferent signaling, which may ultimately compromise baroreflex-related cardiac autonomic regulation [8,23]. The observed association between TAB-BRA and log LF may be compatible with the involvement of baroreflex-related autonomic modulation. However, because BRS was not directly measured in the present study, this mechanistic interpretation remains inferential. Further studies incorporating BRS measurements are needed to test this hypothesis.

To our knowledge, this is the first study to demonstrate the incremental explanatory value of CTA-assessed aortic arch atherosclerotic burden beyond carotid sinus assessment alone in relation to cardiac autonomic dysfunction. Owing to the deep mediastinal location of the aortic arch, previous studies of vascular–autonomic interactions have largely relied on indirect markers, such as pulse wave velocity [24], arterial distensibility [25], and calcification [26], to estimate atherosclerotic burden. Using non-invasive CTA with cross-sectional reconstructions perpendicular to the vessel centerline, the present study directly quantified circumferential plaque involvement along the aortic arch, providing novel evidence of the association between the aortic arch atherosclerotic burden and cardiac autonomic dysfunction. Although the standardized associations of AB-AA and AB-CS with SDNN did not differ significantly, the incremental explanatory value of AB-AA beyond AB-CS may partly reflect physiological differences between the two baroreceptor regions, as aortic baroreceptors have been shown to exhibit greater mechanosensitivity than carotid baroreceptors [27]. These findings underscore the importance of assessing atherosclerotic burden across both the carotid sinus and aortic arch when investigating vascular correlates of post-stroke cardiac autonomic function. Routine CTA with integrated coverage of the carotid arteries and aortic arch provides a feasible, non-invasive approach for the simultaneous assessment of atherosclerotic burden across the entire BRA vascular bed. Given the potential prognostic value of HRV for recurrent vascular events after ischemic stroke, future prospective studies should investigate whether integrating CTA-based BRA atherosclerotic burden assessment with HRV evaluation could improve risk stratification for these events.

The linear regression estimates should be interpreted as overall estimates across the observed TAB-BRA range, rather than as a complete representation of the underlying dose–response pattern. Using restricted cubic splines, we identified a nonlinear inverse association between TAB-BRA and 24 h SDNN, with a steeper decline at lower scores followed by a more gradual but continued decline at higher scores. Nasr et al. [8] reported that BRS was reduced in bilateral, rather than unilateral, carotid atherosclerosis. This reduction was already evident in mild bilateral lesions, with little additional impairment at greater stenosis severity. Although differences in vascular assessment and autonomic outcomes limit direct comparison, both studies identified a nonlinear association between atherosclerotic severity and cardiac autonomic dysfunction. In our study, the steeper decline in SDNN at lower TAB-BRA scores highlights the potential relevance of even a modest BRA atherosclerotic burden to cardiac autonomic dysfunction after ischemic stroke, suggesting that attention to autonomic impairment should extend beyond patients with the highest atherosclerotic burden.

Subgroup analyses in this study suggested potential effect modification by age, with each 1-point increase in TAB-BRA associated with a greater reduction in 24 h SDNN among patients younger than 60 years than among those aged 60 years or older. The comparatively weaker association observed among older patients may be partly attributable to a more generalized age-related decline in autonomic function [28] and cumulative autonomic impairment associated with prolonged exposure to vascular risk factors [29], both of which may obscure the association between BRA atherosclerotic burden and SDNN. The stronger inverse association between TAB-BRA and SDNN among younger patients, together with the steeper decline in SDNN across the lower TAB-BRA range in the RCS analysis, raises the question of whether slowing the progression of atherosclerosis—particularly through the early prevention and management of atherosclerosis in the carotid sinus and aortic arch—could delay the development of associated cardiac autonomic dysfunction. This hypothesis warrants further investigation in future studies.

In addition to its overall level, HRV exhibits a pronounced circadian rhythm reflecting circadian variations in autonomic tone, typically characterized by enhanced parasympathetic dominance and higher HRV during nighttime [11,12]. Cosinor models [15] have recently been applied in HRV analysis to parametrically describe the rhythm-adjusted mean, amplitude, and peak time, thereby preserving richer temporal information than traditional day–night segmented HRV metrics. In the present study, the SDNN MESOR decreased progressively with increasing TAB-BRA, mirroring the decline observed for 24 h levels. Although SDNN amplitude did not exhibit a strictly linear reduction across continuous TAB-BRA values, amplitudes in both Tertile 2 and Tertile 3 were significantly lower than in Tertile 1. These findings suggest that circadian SDNN oscillation is attenuated in patients with moderate-to-high atherosclerotic burden in the BRAs. Baroreflex-related mechanisms may be considered as one possible explanation for this finding. Animal studies have shown that sinoaortic denervation markedly disrupts the normal circadian rhythm of blood pressure [30]. Furthermore, Pelat et al. observed disrupted circadian rhythms of heart rate and systolic blood pressure, accompanied by reduced HRV, in a mouse model of atherosclerosis [31]. In line with these findings, our previous work [10] demonstrated that higher TAB-BRA was closely associated with blunted nocturnal blood pressure dipping in post-stroke patients; such a non-dipping pattern has also been associated with reduced circadian variation in HRV [32]. Taken together, these observations raise the possibility that BRA atherosclerosis, potentially through baroreflex dysfunction and altered circadian blood pressure regulation, may contribute to the attenuated circadian amplitude observed in HRV. Nevertheless, BRS and its circadian variation were not measured concurrently in this study, and prospective investigations incorporating simultaneous assessments of HRV, ambulatory blood pressure, and baroreflex function are needed to test this putative mechanism.

This study has the following strengths. First, TAB-BRA simultaneously quantifies the extent of atherosclerotic involvement in the aortic arch and carotid sinus and therefore characterizes BRA lesions more completely than assessment of a single vascular bed or stenosis severity alone. Second, the study population consisted predominantly of patients with minor stroke with a median NIHSS score of 2, the median interval from stroke onset to Holter monitoring was 7 days, and the clinical condition had been stable for the 3 days before monitoring, which helped reduce interference from stroke-related stress and clinical fluctuations in the assessment of autonomic function. Finally, using segment-level HRV parameters measured repeatedly over 24 h, this study applied a mixed-effects cosinor model to characterize post-stroke circadian autonomic dysregulation, providing a more comprehensive assessment than simple day–night grouping by preserving the temporal structure of the full 24 h profile and directly estimating key rhythm parameters. By focusing on BRA atherosclerotic burden, this study provides a peripheral vascular perspective for understanding the abnormal circadian regulation of HRV after ischemic stroke.

This study also has several limitations. First, because this was a cross-sectional study, causal and pathophysiological relationships cannot be established and require confirmation in future studies. Second, BRS was not directly measured. Therefore, whether baroreflex dysfunction contributes to the association between TAB-BRA and HRV remains uncertain and warrants direct investigation. Third, although 24 h Holter monitoring can reflect autonomic function under patients’ usual conditions, HRV indices are inevitably affected by changes in posture and physical activity. Owing to practical constraints, these factors were not systematically assessed in this study. Nevertheless, all participants were managed under standardized conditions in the stroke center wards, with relatively uniform routines and environments, which reduced confounding from extreme external factors to some extent. Fourth, sleep-related factors, particularly obstructive sleep apnea (OSA), which is highly prevalent among patients with stroke, may substantially affect cardiac autonomic regulation and potentially alter the circadian rhythm of HRV [33]. However, the diagnosis of OSA generally requires sleep monitoring, which was not routinely performed in the present study. Future studies should systematically assess OSA and sleep conditions and account for their potential confounding effects. Finally, this was a single-center study with a relatively limited sample, and the study population was restricted to patients with ischemic stroke, limiting the physiological interpretation and generalization of the findings to non-stroke populations. Multicenter, large-sample cohort studies including healthy individuals are still needed to further verify the stability and clinical value of these findings.

## 5. Conclusions

Among patients with ischemic stroke, the nonlinear inverse association between TAB-BRA and 24 h SDNN was characterized by a steeper decrease in SDNN at lower TAB-BRA values. This pattern suggests that measurable impairment of cardiac autonomic regulation may be present even when atherosclerotic involvement of the baroreceptor-resident arteries is relatively limited and may therefore merit attention during cardiac autonomic assessment. The attenuated circadian SDNN amplitudes in the middle and highest TAB-BRA tertiles further indicate blunted day–night variation in cardiac autonomic regulation at higher levels of atherosclerotic burden. Aortic arch atherosclerotic burden provided additional explanatory value beyond carotid sinus burden and therefore warrants attention when investigating vascular correlates of post-stroke cardiac autonomic dysfunction. Future studies are warranted to clarify the potential contribution of baroreflex dysfunction to the association between BRA atherosclerosis and HRV, and to investigate whether BRA atherosclerosis-associated autonomic dysfunction contributes to recurrent cardiovascular events after ischemic stroke.

## Figures and Tables

**Table 1:** Baseline characteristics and 24 h HRV parameters of enrolled patients by tertiles of TAB-BRA.

Variable	Tertile 1 (*n* = 75)	Tertile 2 (*n* = 85)	Tertile 3 (*n* = 93)	Overall *p*
Age, years, M (IQR)	50 (39, 57)	60 (54, 65) ***	66 (59, 71) ***	<0.001
Sex				0.411
Male, *n* (%)	59 (78.7)	59 (69.4)	69 (74.2)	
Female, *n* (%)	16 (21.3)	26 (30.6)	24 (25.8)	
Time from stroke onset to admission, days, M (IQR)	4 (2, 7)	3 (2, 6)	3 (2, 7)	0.740
Time from stroke onset to Holter monitoring, days, M (IQR)	7 (5, 13)	7 (5, 10)	8 (5, 11)	0.580
BMI, kg/m^2^, M (IQR)	26.0 (23.5, 29.2)	24.8 (22.9, 27.1)	25.4 (23.7, 27.7)	0.182
Hypertension, *n* (%)	33 (44.0)	57 (67.1) **	59 (63.4) *	0.007
Diabetes mellitus, *n* (%)	21 (28.0)	27 (31.8)	37 (39.8)	0.249
History of coronary artery disease, *n* (%)	2 (2.7)	8 (9.4)	10 (10.8)	0.127
Current or previous smoking, *n* (%)	29 (38.7)	31 (36.5)	52 (55.9) *	0.017
Glucose, mmol/L, M (IQR)	4.96 (4.54, 6.40)	5.02 (4.48, 7.16)	5.31 (4.75, 6.79)	0.092
Total cholesterol, mmol/L, M (IQR)	4.10 (3.62, 4.52)	4.05 (3.47, 4.78)	3.96 (3.39, 4.63)	0.760
HDL-C, mmol/L, M (IQR)	1.03 (0.87, 1.23)	1.03 (0.84, 1.20)	1.03 (0.89, 1.18)	0.795
LDL-C, mmol/L, M (IQR)	2.45 (1.88, 2.88)	2.48 (1.87, 2.90)	2.39 (1.78, 2.86)	0.757
Systolic blood pressure, mmHg, M (IQR)	140 (127, 155)	141 (130, 157)	147 (134, 164) *	0.025
Diastolic blood pressure, mmHg, M (IQR)	92 (83, 102)	90 (83, 98)	88 (80, 98)	0.366
Admission NIHSS, M (IQR)	2 (1, 3)	2 (1, 4)	2 (1, 4)	0.783
Insula involvement, *n* (%)	4 (5.3)	7 (8.2)	5 (5.4)	0.674
Brainstem involvement, *n* (%)	21 (28.0)	21 (24.7)	28 (30.1)	0.721
Infarct territory				0.295
Anterior circulation, *n* (%)	43 (57.3)	55 (64.7)	54 (58.1)	
Posterior circulation, *n* (%)	32 (42.7)	30 (35.3)	36 (38.7)	
Anterior and posterior circulation, *n* (%)	0 (0.0)	0 (0.0)	3 (3.2)	
SDNN, ms, M (IQR)	117.0 (101.5, 141.0)	106.0 (83.0, 125.0) **	93.0 (77.0, 110.0) ***	<0.001
rMSSD, ms, M (IQR)	28.0 (23.0, 36.0)	25.0 (19.0, 33.0) **	25.0 (19.0, 33.0) *	0.019
LF, ms^2^, M (IQR)	471.9 (276.4, 650.8)	282.5 (178.4, 422.6) ***	249.2 (115.6, 392.9) ***	<0.001
HF, ms^2^, M (IQR)	145.6 (99.3, 255.3)	111.0 (57.5, 170.8) ***	96.7 (65.0, 168.1) ***	<0.001

Data are presented as median (IQR) or *n* (%). Overall *p* values compare the three TAB-BRA tertiles. Superscripts indicate comparisons with Tertile 1: * *p* < 0.05, ** *p* < 0.01, and *** *p* < 0.001. Abbreviations: M, median; IQR, interquartile range; TAB-BRA, total atherosclerosis burden of baroreceptor-resident arteries; HRV, heart rate variability; BMI, body mass index; HDL-C, high-density lipoprotein cholesterol; LDL-C, low-density lipoprotein cholesterol; NIHSS, National Institutes of Health Stroke Scale; SDNN, standard deviation of all normal-to-normal intervals; rMSSD, root mean square of successive differences between adjacent normal-to-normal intervals; LF, low-frequency power; HF, high-frequency power.

**Table 2:** Associations between TAB-BRA and HRV parameters in linear regression analyses.

24 H HRV Parameters	Unadjusted Model		Adjusted Model *	
	Crude β (95% CI)	*p* Value	Adjusted β (95% CI)	*p* Value
SDNN, ms	−1.81 (−2.46 to −1.15)	<0.001	−1.67 (−2.43 to −0.90)	<0.001
rMSSD, ms	−0.22 (−0.49 to 0.06)	0.118	−0.09 (−0.43 to 0.24)	0.595
log LF ^†^	−0.06 (−0.08 to −0.05)	<0.001	−0.05 (−0.07 to −0.03)	<0.001
log HF ^†^	−0.03 (−0.05 to −0.01)	0.001	−0.01 (−0.03 to 0.01)	0.427

* Adjusted for age, sex, hypertension, diabetes mellitus, body mass index, history of coronary artery disease, smoking, time from stroke onset to Holter monitoring, National Institutes of Health Stroke Scale, insular involvement, and brainstem involvement. ^†^ For natural log-transformed LF and HF, regression coefficients can be interpreted as percentage differences in geometric means using (exp(β) − 1) × 100%. For example, an adjusted β of −0.05 for log LF indicates that each 1-point higher TAB-BRA score was associated with an approximately 4.9% lower geometric mean LF. Abbreviations: TAB-BRA, total atherosclerosis burden of baroreceptor-resident arteries; CI, confidence interval; HRV, heart rate variability; SDNN, standard deviation of all normal-to-normal intervals; rMSSD, root mean square of successive differences between adjacent normal-to-normal intervals; LF, low-frequency power; HF, high-frequency power.

**Table 3:** Incremental explanatory value of atherosclerotic burden of the aortic arch beyond carotid sinus burden for 24 h HRV parameters.

24 H HRV Parameter	AB-CS Model *	AB-CS and AB-AA Model ^†^	Adjusted R^2^ Difference	*p* for Difference
	Adjusted R^2^	Adjusted R^2^		
SDNN	0.161	0.194	0.033	0.001
rMSSD	0.013	0.028	0.015	0.032
log LF	0.263	0.272	0.009	0.053
log HF	0.103	0.115	0.012	0.040

All analyses included 253 patients. * The AB-CS model included AB-CS and age, sex, hypertension, diabetes mellitus, body mass index, history of coronary artery disease, smoking, time from stroke onset to Holter monitoring, National Institutes of Health Stroke Scale, insular involvement, and brainstem involvement. ^†^ The AB-CS and AB-AA model additionally included AB-AA. Abbreviations: AB-AA, atherosclerotic burden of the aortic arch; AB-CS, atherosclerotic burden of the carotid sinus; HRV, heart rate variability; SDNN, standard deviation of all normal-to-normal intervals; rMSSD, root mean square of successive differences between adjacent normal-to-normal intervals; LF, low-frequency power; HF, high-frequency power.

**Table 4:** Site-specific associations of the atherosclerotic burden of the aortic arch and carotid sinus with 24 h HRV parameters.

24 H HRV Parameters	AB-AA Adjusted β (95% CI)	AB-CS Adjusted β (95% CI)	AB-AA − AB-CS β difference (95% CI)	*p* for Difference
SDNN, ms	−8.31 (−13.25 to −3.36)	−2.82 (−7.62 to 1.99)	−5.49 (−13.93 to 2.95)	0.201
rMSSD, ms	−2.35 (−4.49 to −0.20)	1.46 (−0.63 to 3.54)	−3.80 (−7.46 to −0.14)	0.042
log LF	−0.13 (−0.25 to 0.00)	−0.18 (−0.30 to −0.06)	0.06 (−0.16 to 0.27)	0.609
log HF	−0.15 (−0.28 to −0.01)	0.07 (−0.06 to 0.21)	−0.22 (−0.45 to 0.02)	0.073

Site-specific atherosclerotic burden scores were standardized as Z scores and entered simultaneously into the multivariable linear regression model. The β difference was calculated as β(AB-AA) − β(AB-CS). Models were adjusted for age, sex, hypertension, diabetes mellitus, body mass index, history of coronary artery disease, smoking, time from stroke onset to Holter monitoring, National Institutes of Health Stroke Scale, insular involvement, and brainstem involvement. Abbreviations: AB-AA, atherosclerotic burden of the aortic arch; AB-CS, atherosclerotic burden of the carotid sinus; HRV, heart rate variability; CI, confidence interval; SDNN, standard deviation of all normal-to-normal intervals; rMSSD, root mean square of successive differences between adjacent normal-to-normal intervals; LF, low-frequency power; HF, high-frequency power.

**Table 5:** Associations between TAB-BRA and circadian rhythm parameters of SDNN.

TAB-BRA as a Continuous Variable				
Rhythm Parameter		Adjusted β (95% CI)		*p* Value
MESOR, ms		−0.70 (−1.04 to −0.34)		<0.001
Amplitude, ms		−0.18 (−0.38 to 0.03)		0.096
Peak time, h *		−0.04 (−0.09 to 0.01)		0.084
**TAB-BRA Categorized into Tertiles**				
**Rhythm Parameter**	**TAB-BRA Tertile**	**Adjusted Estimate (95% CI)**	**Difference (95% CI)**	***p* Value**
MESOR, ms	Tertile 1	53.00 (47.37 to 58.52)	Reference	— ^†^
	Tertile 2	48.57 (43.58 to 53.53)	−4.43 (−9.14 to 0.01)	0.057
	Tertile 3	43.56 (38.40 to 48.45)	−9.44 (−14.54 to −4.27)	<0.001
Amplitude, ms	Tertile 1	8.60 (5.39 to 11.96)	Reference	— ^†^
	Tertile 2	5.00 (2.12 to 8.04)	−3.61 (−6.33 to −0.81)	0.006
	Tertile 3	4.90 (2.03 to 8.00)	−3.71 (−6.59 to −0.77)	0.012
Peak time, h *	Tertile 1	3.03 (2.24 to 3.91)	Reference	— ^†^
	Tertile 2	2.80 (1.34 to 4.31)	−0.23 (−1.39 to 0.89)	0.632
	Tertile 3	3.29 (1.93 to 5.10)	0.26 (−0.88 to 1.74)	0.648

* For peak time, negative and positive differences indicate earlier and later fitted peaks, respectively. ^†^ *p* value for Tertile 1 is not applicable, as it served as the reference group. Models were adjusted for age, sex, hypertension, diabetes mellitus, body mass index, history of coronary artery disease, smoking, time from stroke onset to Holter monitoring, National Institutes of Health Stroke Scale, insular involvement, and brainstem involvement. Abbreviations: TAB-BRA, total atherosclerosis burden of baroreceptor-resident arteries; CI, confidence interval; MESOR, midline estimating statistic of rhythm; SDNN, standard deviation of all normal-to-normal intervals.

## Data Availability

The data presented in this study are available on request from the corresponding author due to privacy and ethical concerns.

## Supplementary Materials

The following supporting information can be downloaded at: https://www.mdpi.com/article/10.3390/biomedicines14102376/s1, Table S1. Heart rate variability indices during the 24 h, daytime, and nighttime periods according to tertiles of TAB-BRA. Table S2. Associations of daytime and nighttime heart rate variability parameters with TAB-BRA in linear regression analyses. Table S3. Sensitivity analysis of the associations between TAB-BRA and 24 h HRV indices with systolic blood pressure replacing hypertension status. Table S4. Associations of AB-AA and AB-CS with 24 h heart rate variability parameters in linear regression analyses. Table S5. AIC-based model fit comparison of the AB-CS model and the combined AB-CS and AB-AA model for 24 h HRV parameters. Table S6. Variance inflation factors for variables in the multivariable model simultaneously including AB-AA and AB-CS. Table S7. Associations between TAB-BRA and circadian rhythm parameters of log LF. Table S8. Associations between TAB-BRA and circadian rhythm parameters of rMSSD. Table S9. Associations between TAB-BRA and circadian rhythm parameters of log HF. Figure S1. Multivariable-adjusted fitted rhythm curves of rMSSD and log HF by TAB-BRA tertiles.

## Abbreviations

The following abbreviations are used in this manuscript:

TAB-BRA	Total atherosclerosis burden of baroreceptor-resident arteries
HRV	Heart rate variability
SDNN	Standard deviation of all normal-to-normal intervals
LF	Low-frequency power
HF	High-frequency power
rMSSD	Root mean square of successive differences between adjacent normal-to-normal intervals
AA	Aortic arch
CS	Carotid sinus
AB-AA	Atherosclerotic burden of the aortic arch
AB-CS	Atherosclerotic burden of the carotid sinus
CT	Computed tomography
MRI	Magnetic resonance imaging
CTA	Computed tomography angiography
BMI	Body mass index
BRA	Baroreceptor-resident artery
BRS	Baroreflex sensitivity
SD	Standard deviation
CI	Confidence interval
HDL-C	High-density lipoprotein cholesterol
IQR	Interquartile range
LDL-C	Low-density lipoprotein cholesterol
MESOR	Midline estimating statistic of rhythm
NIHSS	National Institutes of Health Stroke Scale
RCS	Restricted cubic spline
NN	Normal-to-normal
ICC	Intraclass correlation coefficient
AIC	Akaike information criterion
OSA	Obstructive sleep apnea
