# Supplementary material for: Association of Total Atherosclerosis Burden of Baroreceptor-Resident Arteries with Heart Rate Variability and Its Circadian Rhythm in Patients with Ischemic Stroke
Source: Biomedicines. 2026 Oct 18;14(10):2376. doi: 10.3390/biomedicines14102376 (PMC13702135; doi:10.3390/biomedicines14102376)
Supplement: Supplementary file 1 [file biomedicines-14-02376-s001.zip › biomedicines-4567453-supplementary.pdf]

Supplementary Material

Table S1. Heart rate variability indices during the 24 h, daytime, and nighttime periods according to tertiles of TAB-BRA

HRV Parameter	Tertile 1 (N = 75)	Tertile 2 (N = 85)	Tertile 3 (N = 93)	Overall *p*	*p* for trend*
24 h					
SDNN, ms, M (IQR)	117.0 (101.5, 141.0)	106.0 (83.0, 125.0)	93.0 (77.0, 110.0)	< 0.001	< 0.001
rMSSD, ms, M (IQR)	28.0 (23.0, 36.0)	25.0 (19.0, 33.0)	25.0 (19.0, 33.0)	0.019	0.033
LF, ms^2, M (IQR)	471.9 (276.4, 650.8)	282.5 (178.4, 422.6)	249.2 (115.6, 392.9)	< 0.001	< 0.001
HF, ms^2, M (IQR)	145.6 (99.3, 255.3)	111.0 (57.50, 170.8)	96.7 (65.0, 168.1)	< 0.001	< 0.001
Daytime (6:00-22:00)					
SDNN, ms, M (IQR)	90 (79.5,111.5)	87 (66.0, 104.0)	75.0 (65.0, 91.0)	0.001	< 0.001
rMSSD, ms, M (IQR)	26 (21.5, 33.0)	23.0 (18.0, 32.0)	24 (19.0, 32.0)	0.200	0.179
LF, ms^2, M (IQR)	391.8 (240.2, 561.9)	247.8 (151.8, 393.1)	204.5 (116.5, 296.7)	< 0.001	< 0.001
HF, ms^2, M (IQR)	128.9 (73.2, 205.0)	89 (50.4, 153.5)	80.20 (52.0, 137.6)	0.004	0.003
Nighttime (22:00-6:00)					
SDNN, ms, M (IQR)	97.0 (82.0, 115.0)	93.0 (68.0, 113.0)	81.0 (64.0, 95.0)	< 0.001	< 0.001
rMSSD, ms, M (IQR)	33.0 (26.5, 42.0)	26.0 (21.0, 34.0)	27.0 (21.0, 34.0)	< 0.001	0.003
LF, ms^2, M (IQR)	569.1 (350.8, 912.1)	304.2 (176.0, 496.5)	273.7 (118.2, 436.0)	< 0.001	< 0.001
HF, ms^2, M (IQR)	183.6 (140.3, 389.6)	132.5 (80.3, 234.1)	111.8 (69.4, 200.8)	< 0.001	< 0.001

Data are presented as median (IQR). **p* for trend was calculated using the Jonckheere–Terpstra trend test. Abbreviations: HRV, heart rate variability; SDNN, standard deviation of all normal-to-normal intervals; rMSSD, root mean square of successive differences between adjacent normal-to-normal intervals; LF, low-frequency power; HF, high-frequency power; M, median; IQR, Interquartile Range.

Table S2. Associations of daytime and nighttime heart rate variability parameters with TAB-BRA in linear regression analyses

HRV parameter	Unadjusted model		Adjusted model *	
	β (95% CI)	*p*	β (95% CI)	*p*
Daytime (6:00-22:00)				
SDNN, ms	−1.13(−1.70 to −0.55)	< 0.001	−1.15(−1.83 to −0.47)	0.001
rMSSD, ms	−0.11(−0.38 to 0.16)	0.430	−0.08(−0.41 to 0.25)	0.646
log LF†	−0.06 (−0.08 to −0.04)	< 0.001	−0.04 (−0.06 to −0.02)	< 0.001
log HF†	−0.022 (−0.04 to 0.00)	0.017	−0.01 (−0.03 to 0.02)	0.574
Nighttime (22:00-6:00)				
SDNN, ms	−1.52(−2.11 to −0.93)	< 0.001	−1.44(−2.15 to −0.73)	< 0.001
rMSSD, ms	−0.41(−0.72 to −0.11)	0.008	−0.14(−0.51 to 0.24)	0.471
log LF†	−0.07 (−0.09 to −0.05)	< 0.001	−0.06 (−0.08 to −0.04)	< 0.001
log HF†	−0.04 (−0.06 to −0.02)	< 0.001	−0.01 (−0.04 to 0.01)	0.211

*Adjusted for age, sex, hypertension, diabetes mellitus, body mass index, history of coronary artery disease, smoking, time from stroke onset to Holter monitoring, National Institutes of Health Stroke Scale, insular involvement, and brainstem involvement. † For natural log-transformed LF and HF, regression coefficients can be interpreted as percentage differences in geometric means using (exp(β) − 1) × 100%. Abbreviations: TAB-BRA, total atherosclerosis burden of baroreceptor-resident arteries; CI, confidence interval; HRV, heart rate variability; SDNN, standard deviation of all normal-to-normal intervals; rMSSD, root mean square of successive differences between adjacent normal-to-normal intervals; LF, low-frequency power; HF, high-frequency power.

Table S3. Sensitivity analysis of the associations between TAB-BRA and 24 h HRV indices with systolic blood pressure replacing hypertension status.

**24 H HRV Parameter**	**Adjusted model ***	
	**β (95% CI)**	***p Value***
SDNN, ms	−1.62 (−2.39 to −0.85)	< 0.001
rMSSD, ms	−0.08 (−0.41 to 0.26)	0.642
log LF	−0.05 (−0.07 to −0.03)	< 0.001
log HF	−0.01 (−0.03 to 0.01)	0.515

*Adjusted for age, sex, systolic blood pressure, diabetes mellitus, body mass index, history of coronary artery disease, smoking, time from stroke onset to Holter monitoring, National Institutes of Health Stroke Scale, insular involvement, and brainstem involvement. Abbreviations: TAB-BRA, total atherosclerosis burden of baroreceptor-resident arteries; CI, confidence interval; HRV, heart rate variability; SDNN, standard deviation of all normal-to-normal intervals; rMSSD, root mean square of successive differences between adjacent normal-to-normal intervals; LF, low-frequency power; HF, high-frequency power.

Table S4. Associations of AB-AA and AB-CS with 24 h heart rate variability parameters in linear regression analyses.

**AB-AA**				
**HRV parameter**	**Crude β (95% CI)**	***p***	**Adjusted β (95% CI)***	***p***
SDNN, ms	−4.20 (−5.65 to −2.75)	< 0.001	−3.81 (−5.48 to −2.13)	< 0.001
rMSSD, ms	−0.82 (−1.42 to −0.22)	0.008	−0.62 (−1.35 to 0.10)	0.092
log LF	−0.13 (−0.17 to −0.10)	< 0.001	−0.09 (−0.13 to −0.04)	< 0.001
log HF	−0.08 (−0.12 to −0.04)	< 0.001	−0.04 (−0.09 to 0.00)	0.077
**AB-CS**				
**HRV parameter**	**Crude β (95% CI)**	***p***	**Adjusted β (95% CI)***	***p***
SDNN, ms	−2.23 (−3.25 to −1.22)	< 0.001	−1.83 (−2.96 to −0.69)	0.002
rMSSD, ms	−0.12 (−0.53 to 0.30)	0.577	0.09 (−0.40 to 0.57)	0.730
log LF	−0.08 (−0.11 to −0.06)	< 0.001	−0.06 (−0.09 to −0.04)	< 0.001
log HF	−0.03 (−0.06 to 0.00)	0.041	0.00 (−0.03 to 0.03)	0.984

*Adjusted for age, sex, hypertension, diabetes mellitus, body mass index, history of coronary artery disease, smoking, time from stroke onset to Holter monitoring, National Institutes of Health Stroke Scale, insular involvement, and brainstem involvement. Abbreviations: CI, confidence interval; AB-AA, atherosclerotic burden of the aortic arch; AB-CS, atherosclerotic burden of the carotid sinus; HRV, heart rate variability; SDNN, standard deviation of all normal-to-normal intervals; rMSSD, root mean square of successive differences between adjacent normal-to-normal intervals; LF, low-frequency power; HF, high-frequency power.

Table S5. AIC-based model fit comparison of the AB-CS model and the combined AB-CS and AB-AA model for 24 h HRV parameters.

24 H HRV Parameter	AB-CS Model*	AB-CS and AB-AA Model†	Δ AIC‡
	AIC	AIC	
SDNN	2438.04	2428.71	−9.32
rMSSD	2008.55	2005.68	−2.87
log LF	576.20	574.22	−1.98
log HF	621.85	619.40	−2.46

* The AB-CS model included AB-CS and age, sex, hypertension, diabetes mellitus, body mass index, history of coronary artery disease, smoking, time from stroke onset to Holter monitoring, National Institutes of Health Stroke Scale, insular involvement, and brainstem involvement. † The AB-CS + AB-AA model additionally included AB-AA. ‡ ΔAIC was calculated as AIC of the AB-CS + AB-AA model minus AIC of the AB-CS model, negative values indicate better fit of the combined model. AB-AA, atherosclerotic burden of the aortic arch; AB-CS, atherosclerotic burden of the carotid sinus; AIC, Akaike information criterion; HRV, heart rate variability; SDNN, standard deviation of all normal-to-normal intervals; rMSSD, root mean square of successive differences between adjacent normal-to-normal intervals; LF, low-frequency power; HF, high-frequency power.

Table S6. Variance inflation factors for variables in the multivariable model simultaneously including AB-AA and AB-CS

Variable	VIF
AB-CS, standardized	1.844
AB-AA, standardized	1.956
Age	1.665
Sex	1.359
Hypertension	1.130
Diabetes mellitus	1.054
Body mass index	1.184
History of coronary artery disease	1.140
Smoking	1.385
Time from onset to Holter monitoring	1.085
NIHSS score	1.169
Insular involvement	1.225
Brainstem involvement	1.113

AB-AA and AB-CS scores were standardized as Z scores and simultaneously entered into the multivariable linear regression model. Multivariable linear regression model included standardized AB-CS, standardized AB-AA, age, sex, hypertension, diabetes mellitus, body mass index, history of coronary artery disease, smoking, time from stroke onset to Holter monitoring, National Institutes of Health Stroke Scale, insular involvement, and brainstem involvement. Abbreviations: AB-AA, atherosclerotic burden of the aortic arch; AB-CS, atherosclerotic burden of the carotid sinus; VIF, variance inflation factor; NIHSS, National Institutes of Health Stroke Scale.

Table S7. Associations between TAB-BRA and circadian rhythm parameters of log LF

TAB-BRA as a continuous variable		
**Rhythm parameter**	**Adjusted β (95% CI)***	***p* value**
MESOR	−0.05 (−0.07 to −0.03)	< 0.001
Amplitude	−0.01 (−0.02 to 0.00)	0.108
Peak time, h†	−0.12 (−0.28 to 0.04)	0.137

TAB-BRA categorized into tertiles				
**Rhythm parameter**	**TAB-BRA tertile**	**Adjusted estimate (95% CI)***	**Difference (95% CI)**	***p* value**
**MESOR**	Tertile 1	5.69 (5.36 to 6.01)	Reference	—#
	Tertile 2	5.49 (5.20 to 5.76)	−0.19 (−0.46 to 0.07)	0.154
	Tertile 3	5.20 (4.90 to 5.48)	−0.49 (−0.79 to −0.20)	0.002
**Amplitude**	Tertile 1	0.24 (0.10 to 0.38)	Reference	—#
	Tertile 2	0.11 (0.03 to 0.24)	−0.13 (−0.24 to 0.00)	0.037
	Tertile 3	0.12 (0.02 to 0.25)	−0.12 (−0.24 to 0.02)	0.073
**Peak time, h†**	Tertile 1	1.91 (23.90 to 24.00/0.00 to 3.51) ‡	Reference	—#
	Tertile 2	1.21 (18.46 to 24.00/0.00 to 4.88) ‡	−0.70 (−5.14 to 3.75)	0.759
	Tertile 3	3.53 (23.14 to 24.00/ 0.00 to 10.01) ‡	1.62 (−3.09 to 6.32)	0.501

*Models adjusted for age, sex, hypertension, diabetes mellitus, body mass index, history of coronary artery disease, smoking, time from stroke onset to Holter monitoring, National Institutes of Health Stroke Scale, insular involvement, and brainstem involvement. †Negative and positive peak-time differences indicate earlier and later peaks, respectively. ‡ A split peak-time CI crosses midnight. # *p* value for Tertile 1 is not applicable, as it served as the reference group. Abbreviations: MESOR, midline estimating statistic of rhythm; TAB-BRA, total atherosclerosis burden of baroreceptor-resident arteries; LF, low-frequency power; CI, confidence interval.

Table S8. Associations between TAB-BRA and circadian rhythm parameters of rMSSD

**TAB-BRA as a continuous variable**		
**Rhythm parameter**	**Adjusted β (95% CI)***	***p* value**
MESOR, ms	−0.09 (−0.42 to 0.24)	0.583
Amplitude, ms	−0.06 (−0.19 to 0.07)	0.369
Peak time, h†	−0.09 (−0.25 to 0.03)	0.210

**TAB-BRA categorized into tertiles**				
**Rhythm parameter**	**TAB-BRA tertile**	**Adjusted estimate (95% CI)***	**Difference (95% CI)**	***p* value**
**MESOR, ms**	Tertile 1	29.63 (24.34 to 34.84)	Reference	—#
	Tertile 2	27.21 (22.50 to 31.76)	−2.42 (−6.81 to 1.71)	0.272
	Tertile 3	27.06 (22.22 to 31.67)	−2.57 (−7.32 to 2.21)	0.265
**Amplitude, ms**	Tertile 1	4.75 (2.75 to 6.93)	Reference	—#
	Tertile 2	1.59 (0.46 to 3.54)	−3.17 (−4.80 to −1.12)	0.002
	Tertile 3	2.80 (0.95 to 4.92)	−1.95 (−3.73 to −0.02)	0.042
**Peak time, h†**	Tertile 1	1.80(0.88 to 2.60)	Reference	—#
	Tertile 2	0.30 (20.61 to 24.00 / 0.00 to 2.73)‡	−1.50(−4.76 to 1.76)	0.367
	Tertile 3	2.25(0.86 to 3.42)	0.45(−1.21 to 2.11)	0.593

*Models adjusted for age, sex, hypertension, diabetes mellitus, body mass index, history of coronary artery disease, smoking, time from stroke onset to Holter monitoring, National Institutes of Health Stroke Scale, insular involvement, and brainstem involvement. †Negative and positive peak-time differences indicate earlier and later peaks, respectively. ‡ A split peak-time CI crosses midnight. # *p* value for Tertile 1 is not applicable, as it served as the reference group. Abbreviations: MESOR, midline estimating statistic of rhythm; TAB-BRA, total atherosclerosis burden of baroreceptor-resident arteries; rMSSD, root mean square of successive differences between adjacent normal-to-normal intervals; CI, confidence interval.

Table S9. Associations between TAB-BRA and circadian rhythm parameters of log HF

TAB-BRA as a continuous variable		
**Rhythm parameter**	**Adjusted β (95% CI)***	***p* value**
MESOR	−0.01 (−0.03 to 0.01)	0.409
Amplitude	−0.01 (−0.02 to 0.00)	0.112
Peak time, h†	−0.09 (−0.18 to 0.01)	0.062

TAB-BRA categorized into tertiles				
**Rhythm parameter**	**TAB-BRA tertile**	**Adjusted estimate (95% CI)***	**Difference (95% CI)**	***p* value**
**MESOR**	Tertile 1	4.82 (4.47 to 5.17)	Reference	—#
	Tertile 2	4.52 (4.20 to 4.81)	−0.30 (−0.60 to −0.02)	0.041
	Tertile 3	4.51 (4.19 to 4.82)	−0.31 (−0.63 to 0.01)	0.058
**Amplitude**	Tertile 1	0.42 (0.28 to 0.57)	Reference	—#
	Tertile 2	0.22 (0.11 to 0.35)	−0.20 (−0.32 to −0.08)	0.001
	Tertile 3	0.27 (0.14 to 0.41)	−0.15 (−0.27 to −0.02)	0.021
**Peak time, h†**	Tertile 1	1.51 (0.36 to 2.49)	Reference	—#
	Tertile 2	1.09 (22.80 to 24.00/0.00 to 2.72) ‡	−0.42 (−2.01 to 1.16)	0.602
	Tertile 3	2.37 (0.82 to 3.88)	0.86 (−0.45 to 2.18)	0.198

*Models adjusted for age, sex, hypertension, diabetes mellitus, body mass index, history of coronary artery disease, smoking, time from stroke onset to Holter monitoring, National Institutes of Health Stroke Scale, insular involvement, and brainstem involvement. †Negative and positive peak-time differences indicate earlier and later peaks, respectively. ‡ A split peak-time CI crosses midnight. # *p* value for Tertile 1 is not applicable, as it served as the reference group. Abbreviations: MESOR, midline estimating statistic of rhythm; TAB-BRA, total atherosclerosis burden of baroreceptor-resident arteries; HF, high-frequency power; CI, confidence interval.

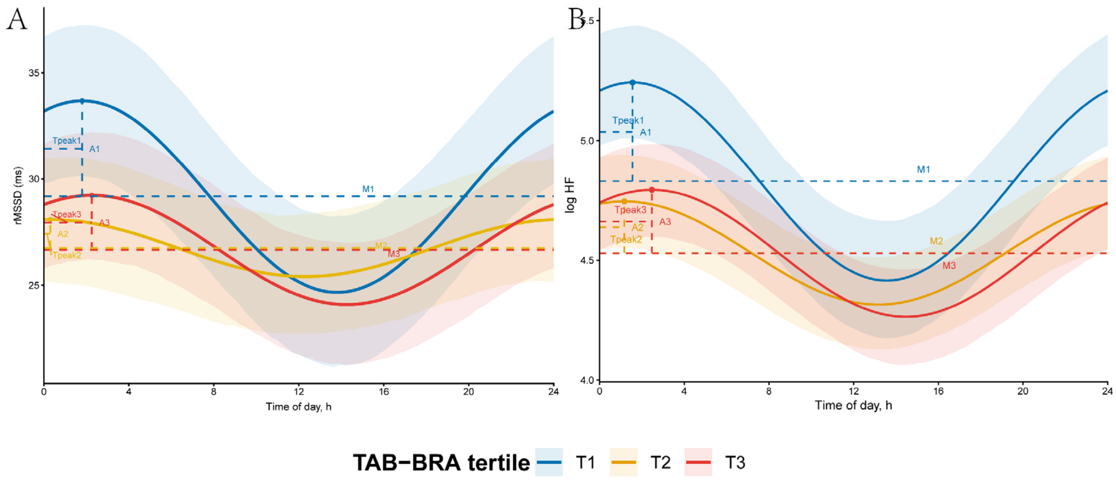

Figure S1. Multivariable-adjusted fitted rhythm curves of rMSSD and log HF by TAB-BRA tertiles. Panels A and B show adjusted 24-hour rhythms of rMSSD and log HF across TAB-BRA Tertiles 1–3, with blue, orange, and red lines representing fitted cosinor curves for Tertiles 1, 2, and 3, respectively, and shaded areas denoting 95% confidence intervals. Long horizontal dashed lines (labeled M1–M3) indicate the MESOR; vertical dashed segments (labeled A1–A3) from the MESOR to the peak indicate amplitude; and the x-coordinates of the peak points (labeled Tpeak1–Tpeak3) indicate peak times for Tertiles 1–3, respectively. Models were adjusted for age, sex, hypertension, diabetes mellitus, body mass index, history of coronary artery disease, smoking, time from stroke onset to Holter monitoring, National Institutes of Health Stroke Scale, insular involvement, and brainstem involvement. Abbreviations: TAB-BRA, total atherosclerosis burden of baroreceptor-resident arteries; rMSSD, root mean square of successive differences between adjacent normal-to-normal intervals; HF, high-frequency power; MESOR, midline estimating statistic of rhythm.
